# Midwifery and the Egg

**Published:** 1974-01

**Authors:** Philip Radford

**Affiliations:** General Practitioner, Bristol


### Midwifery and the Egg

**Philip Radford, M.B., Ch.B., D.C.H.**

General Practitioner, Bristol

While pondering recently on the reasons why a patient of mine was having a somewhat prolonged first stage of labour, I thought, no doubt because I have an interest in natural history, what a sensible method birds have in giving birth to their young. More correctly, of course, the parent birds enable their young to give birth to themselves; I reflected that human midwifery could be managed much more easily if it could incorporate some of the principles of birds' reproductive physiology. In the case of birds the fertilised ovum comes to the outside world in a hard, attractive container complete with its food supply. The egg need only be kept at a suitable temperature for two or three weeks and then the embryo itself cracks its way to the exterior. Naturally, the mother has the effort of laying the egg but this cannot present anything like the hazard of human labour. Antenatal care from fertilised ovum to hatching stage would surely be a simple matter; it should be easy to auscultate through an egg-shell, even though the fetal parts could not be palpated. With the difficulty of determing the presentation and of hearing the fetal heart in really obese mothers, I feel the egg-shell system has much to commend it. True, one would have to ensure that the shell thickness was adequate but ante-laying calcium supplements would help that. Clearly antenatal care of the egg would have to be arranged without too much interference with incubation; chilling would have to be avoided. The eggs of many wild birds are beautifully streaked, blotched and stippled with all varieties of colours. It would be intriguing to speculate on the possible colour combinations one would like to see adopted if man switched to the laying of eggs.

The embryo bird is very helpful in that it ruptures its own membranes before hatching. With such a scheme one would not have to consider whether artificial membrane rupture was indicated; there would be no question whether the fore or hind waters should be broken or if a Drew-Smythe catheter or other instruments should be sterilised in readiness. Several hours before hatching the embryo chick pierces the inner shell membrane with its beak; when this occurs the beak projects into a space containing a gas rich in carbon dioxide and pulmonary respiration starts. Once the lung is ventilated the unhatched bird can call and pre-hatching vocalizations are a feature in several bird species. Just think how useful it would be to hear cries produced by the human fetus at the time of membrane rupture, and how reassuring to the mother! I suppose it is possible, however, that after full dilatation of the cervix and rupture of the membranes the fetus might make some abnormal muscular movement thereby driving air through the glottis and so give a pre-birth cry. Such instances must be very rare and I cannot pretend that I have ever heard such a sound, although my experience of midwifery is limited. Certainly I do not remember reading of a cry of this type in any obstetrical text-book, but amongst birds pre-hatching calls are the rule.

Not content with perforating the inner shell membrane the embryo bird goes on to break the outer shell membrane and the shell by tapping with the egg-tooth of the beak. When the egg is pipped in this way then the chick can breathe pure atmospheric air and peeping noises, or other vocalizations, sound all the louder. As a delightful personal experience, I recall listening to the vigorous calling of a newly-hatched common tern while a nest-mate, still inside a pipped egg, seemed to respond with calls which were hardly of less amplitude; in addition, thin, peeping calls could be heard coming from an unpipped egg. Cries of adult common terns nearby also appeared to stimulate calling by the chicks, whether they were in or outside their shells. Encouragement of fetal progress during labour by soothing words from the mother would be better than the present arrangement; likewise, in the case of multiple pregnancies, it would surely be helpful if a newly-delivered twin could communicate with its fellow still within the uterus.

However, not all sounds heard from an egg in the few hours before hatching are vocal in character. Regular clicking noises occur in some species; one type of clicking is caused by the tapping of the egg-tooth against the shell and a second type is due to rhythmic respiratory movements where the body pulses by muscular action. Such respiratory clicks may be monitored readily by means of a tape-recorder; if respiratory clicks were produced by the newborn infant they would be convenient signals for the paediatrician to record. Apparently there is evidence that in some land birds, such as quails, clicks are utilised in the synchronization of hatching. Presumably the embryos are able to communicate with each other by clicking and by vocalizations; thus they are able to hatch out more or less altogether. As quails lay about ten eggs and the young leave the nest within a few hours of hatching synchronization of emergence is obviously an important matter. Of course, it could hardly be suggested that the hatching synchronization of ten quail eggs bears much relevance to human midwifery. Nevertheless, I have heard of multiple pregnancies following intensive hormone therapy for infertility; maybe even ten fetuses could result when even more potent chemicals have been synthesized.

Certainly, there is much to recommend in the egg system of birth. Also, the hatched young of certain bird species are remarkably independent when compared with the helpless human infant. For instance, the young of many plover and duck species leave the nest soon after hatching; they follow a parent for protection but are able to seek and select their own food items. In this connexion it seems likely that most human parents would prefer to keep to the present method of infant rearing. A precocious neonate grabbing its food from the kitchen soon after return from the labour ward would upset most theories of child psychology. Probably a more acceptable system for humans is where the nestlings are fed by their parents until ready to fly from the nest. It remains to be seen whether obstetricians of the future will dream of attempting to channel the evolution of human parturition towards that of the avian type of organization. But should a present-day obstetrician, perhaps a little impatient with the progress of a mother in labour, feel that he would like to change his patients to the principle of egg-shell hatching he has only one course of action. He should take up poultry breeding.

